# A review of contamination of surface-, ground-, and drinking water in Sweden by perfluoroalkyl and polyfluoroalkyl substances (PFASs)

**DOI:** 10.1007/s13280-016-0848-8

**Published:** 2016-11-14

**Authors:** Stefan Banzhaf, Marko Filipovic, Jeffrey Lewis, Charlotte J. Sparrenbom, Roland Barthel

**Affiliations:** 1 Department of Earth Sciences, University of Gothenburg, Box 460, 405 30 Göteborg, Sweden; 2 NIRAS Sweden AB, Fleminggatan 14, 112 26 Stockholm, Sweden; 3 Tyréns AB, Västra Norrlandsgatan 10B, 903 27 Umeå, Sweden; 4 Department of Geology, Lund University, Sölvegatan 12, 223 62 Lund, Sweden

**Keywords:** AFFF, PFAA, PFAS, PFOS, Firefighting foam, Groundwater

## Abstract

Perfluoroalkyl and polyfluoroalkyl substances (PFASs) are found in aquatic systems, flora, and fauna worldwide. These potentially harmful compounds are also frequently detected in Sweden and have already resulted in severe problems for public drinking water supply, i.e., some wells had to be closed due to high PFAS concentrations both in raw water and produced drinking water. Knowledge on PFAS occurrence in Sweden is still quite low, although monitoring is currently ongoing. This work describes potential sources for PFASs to enter the drinking water supply in Sweden and compares different occurrences of PFASs in raw and drinking water in the country. Moreover, the monitoring history, the legal situation, and remediation actions taken are presented. Finally, future challenges and the way forward in Sweden are discussed.

## Introduction

Perfluoroalkyl and polyfluoroalkyl substances (PFASs) are a group of anthropogenic environmental pollutants that are attracting increasing attention worldwide as they are frequently detected in the aquatic environment, wildlife, and humans (Houde et al. 2011; Post et al. 2012). PFASs have been produced since the 1950s and are used in multiple industrial applications such as water repellent on clothing, leather, cookware, and paper, as well as being surface tension lowering agents in firefighting foam (Prevedouros et al. 2006; Wang et al. 2014). The most studied PFAS subgroup are perfluorinated alkyl acids (PFAAs), which include among others perfluoroalkyl carboxylic acids (PFCAs) and perfluoroalkane sulfonic acids (PFSAs). PFCAs and PFSAs, including their precursor, have shown to be persistent in the environment. Recent studies have shown that some PFASs are toxic for both animals and humans (Borg et al. 2013). Further, the most frequently detected PFASs—perfluorooctane sulfonate (PFOS) and perfluorooctanoic acid (PFOA)—are highly mobile once introduced to the aquatic environment (Fujii et al. 2007), and are not removed by conventional wastewater treatment (Arvaniti and Stasinakis 2015; Filipovic and Berger 2015). They therefore pose a severe threat to clean water supply worldwide (Yan et al. 2015).

Over the last decade, PFASs have been detected in surface- and groundwater worldwide. Both are important sources for drinking water production and as a result public concern has arisen over human exposure risks to PFASs. Several studies have examined the risks associated with PFAS exposure through contaminated food and water (European Food Safety Authority 2012). Major sources of human exposure to PFOA and PFOS include the consumption of fish, meat, and eggs (Vestergren et al. 2012). Drinking water may also be a dominant exposure pathway if the water source is influenced by a PFAS-contaminated source area (Vestergren and Cousins 2009). The Swedish Chemicals Agency has also compiled several reports on PFASs and their use in Sweden, for example, regarding their occurrence in food, makeup, sunscreen, ski wax, clothes, paints, leather, paper, and a lot of other products (Swedish Chemicals Agency 2006, 2012, 2015b).

Legislation can restrict the use of PFASs in different materials in contact with food, i.e., non-stick food packaging supplies and cookware, but the current and past use of PFAS containing aqueous film forming foams (AFFF), the release from PFAS manufacturing industries, and chromium-plating industries will continue to constitute major contaminant sources, i.e., “hotspots” spreading into our drinking water resources. In places where it has already reached the environment a ban is not sufficient, but clean up actions are needed. Legislation has reduced some of the practice, mainly for the use of PFOS, but AFFF-containing other PFASs continue to be used at both military and civilian airfields, as well as by some civilian firefighters (Swedish Chemicals Agency 2015a) and these—together with other potential sources mentioned above—will constitute a major source long into the future. This is also true for most products that contain PFASs: they will continue to leach these chemicals during their lifetime and long after they have been disposed of in landfills. PFAS-contaminated groundwater has been inadvertently used as drinking water supply in Sweden, Germany, the United Kingdom, and the USA among others (Atkinson et al. 2008; Quiñones and Snyder 2009; Gyllenhammar et al. 2015). In order to minimize the human exposure to PFASs, several countries have set guideline values for PFASs in surface water, groundwater, and drinking water supplies, including Sweden (Pettersson et al. 2015; Livsmedelsverket 2016), Germany (Wilhelm et al. 2008), and the UK (Drinking Water Inspectorate 2009).

Reducing the spread of PFASs to and within groundwater in order to minimize human exposure through the consumption of contaminated drinking water is an ongoing societal and technical challenge. Conventional water treatment technologies, such as flocculation, are largely ineffective at removing PFASs. However, established methods exist that can reduce PFAS concentrations in drinking water to acceptable levels, including granular activated carbon (GAC), reverse osmosis, anion-exchange resin, nanofiltration, and electrochemical treatment (Appleman et al. 2014; Schaefer et al. 2015). However, the elimination behavior of many of these methods may be dependent on the carbon chain length of the respective PFAS compound. This is presently a subject of considerable research interest. There are indications, for example, that GAC filtration is effective at removing long chained PFCA with eight or more carbons, including PFOA and PFSA with six or more carbons, including PFHxS, whereas short-chained PFASs cannot be removed as effectively (Eschauzier et al. 2012). As yet, an unsolved problem remains regarding how to efficiently deal with all of the PFASs containing waste materials that are left over by filtration technologies. Electrochemical treatment avoids this problem by breaking down the perfluorinated compounds rather than filtering them out, but this has thus far only been tested at lab scale (Schaefer et al. 2015).

There is a trend within the PFAS manufacturing industry to change from longer perfluorinated chains to shorter ones (Scheringer et al. 2014), as the longer chain substances have attracted attention from both the authorities and the media. The health and environmental effects of using shorter perfluorinated chain substances are, however, still uncertain (Wang et al. 2013). It is becoming increasingly clear that chain length does play a significant role in the behavior of the compound once it has been released into the environment. Shorter chains have been reported to have a higher uptake in lettuce leaves, while longer chain PFASs are found primarily in the roots (Blaine et al. 2014). Furthermore, shorter chain PFASs have been shown to absorb into the liver more readily than those with longer ones which are concentrated in the blood proteins (Lau 2015).

The production and release of PFASs and their replacements are topics of discussion in the scientific community, such as: The *Helsingør statement on poly*- *and perfluorinated alkyl substances* (*PFASs*) by Scheringer et al. (2014), and *the Madrid statement on poly*- *and perfluoroalkyl substances* (*PFASs*) by Blum et al. (2015). One prominent compound of the PFAS group (PFOS) has already been restricted by the European Union (Directive 2006/122/EC). PFOS and its salts are also included in the Stockholm Convention (2015), while PFOS and its derivatives are listed in EU’s Water Directive list of priority substances (Directive 2013/39/EU). A more detailed report on PFASs and risk reduction approaches was recently published by the OECD (2015).

This review article describes potential sources for PFASs to enter the drinking water supply and compares different occurrences of PFASs in both raw and drinking water in Sweden. Moreover, the monitoring history, the legal situation, and remediation actions taken are presented. Finally, future challenges and the way forward in Sweden are discussed.

## PFASs in Swedish raw and drinking water

### Sources

Many different sources have been found to contribute to PFASs present in the environment worldwide, and their relevance varies in different settings. The sources of PFASs to surface- and groundwater can be divided into (i) point and (ii) diffuse sources. A complete screening of all potential sources has not yet been performed anywhere in the world, and examples below are taken from a wide range to highlight potential sources for Sweden.

The most well-studied point sources of PFASs to surface waters are wastewater treatment plants (WWTPs). On a regional scale, a study of several small rivers in Germany concluded that the majority of PFASs entered the rivers via point sources, i.e., municipal WWTPs (Becker et al. 2008). Other point sources, such as industrial emissions from PFAS production sites, were observed to have notable impact on surface water used for tap water production in the USA (Steenland et al. 2013). It has also been noted that high levels of PFASs can be detected in surface waters around commercial and military airfields. This contamination is closely connected with the use of AFFF-containing PFASs (Ahrens et al. 2015; Filipovic et al. 2015b), and their precursors, such as 6:2 FTSA (6:2 fluorotelomer sulfonic acid), which can degrade to short-chain PFASs (Kim et al. 2014). Other potential point sources for PFASs in groundwater are landfills that contain PFAS-contaminated waste. In China, landfill sites were suggested to be a major source of PFASs to groundwater, and that they might pose a risk for tap water contamination (Yan et al. 2015). In Europe, landfill sites have not yet been evaluated to a great extent regarding their PFAS contamination potential for groundwater (Eschauzier et al. 2013), also applicable to Sweden.

Also diffuse sources, i.e., contamination caused from a range of dispersed urban and rural land use activities, such as atmospheric deposition and upstream water input, can be relevant for surface water contamination. This has been tested on larger geographical scales, where mass balance modeling tools have been used to identify the relevance of different input pathways of PFASs into a larger water body. Boulanger et al. (2005) calculated the first mass balance of PFOA on a large lake, including point sources such as WWTPs and diffuse sources such as water inflow. It was concluded that WWTP discharge is a minor input of PFOA to Lake Ontario, compared to inflow from the other Great Lakes. In a similar study, Filipovic et al. (2013) showed a mass balance of PFASs for the Baltic Sea that wet deposition and riverine discharges represented the dominant inputs of PFASs and that discharges by WWTPs from coastal cities were a minor input pathway. In remote areas of Sweden, contamination of groundwater was suggested to originate from rain and snow which are contaminated by diffuse sources (Filipovic et al. 2015a). However, according to Filipovic and Berger (2015), WWTPs still contribute considerable amounts (>5 ng/L) into the aquatic environment. It was also shown that PFAS levels within WWTPs’ effluent can be caused by contributions from either the technosphere, or from both technosphere and tap water, in the case of an affected municipal water source, i.e., recirculation of polluted groundwater into municipal tap water ending up in WWTPs getting discharged into the environment again.

In Sweden, former and current firefighting training areas are considered to be the major (point) source for PFAS contamination of groundwater and surface waters. Other hotspots could also be chemical factories or places where a lot of organic solvents are used. The identified hotspots are mainly concentrated around airports, both those that are commercially operated and those run by The Swedish Armed Forces (Berglind et al. 2013; Ahrens et al. 2015; Filipovic et al. 2015b). Other significant sources were shown to be at the four firefighting training areas (Revinge, Sandö, Rosersberg and Skövde) belonging to the Swedish Civil Contingencies Agency (Swedish Civil Contingencies Agency 2014; Swedish Chemicals Agency 2015c). The Swedish Armed Forces used firefighting foams that contained PFOS between approximately 1985 and 2003 (Borgh 2015). Although the use of these foams was phased out from 2003 to 2008, others that contain a wide range of different fluorinated compounds continue to be used because of their unparalleled effectiveness in eliminating petroleum fires. Furthermore, the tanks of firefighting trucks at some commercial Swedish airports were still contaminated with PFOS in 2010 (Swedish Chemicals Agency 2015c), and decontamination had to be performed in 2011 (Norström et al. 2013) before the European parliament-mandated ban of PFOS came into effect.

Two production facilities of AFFF using PFASs (Helsingborg and Vadstena) have been identified (Swedish Civil Contingencies Agency 2014; Swedish Chemicals Agency 2015c), but to our knowledge there are no studies performed on their impact on workers or the environment.

### Monitoring history

The first environmental screenings on PFASs in Sweden were realized 15 years ago by the Department of Applied Environmental Science (ITM), Stockholm University (Regeringskansliet 2016). However, they did not receive much public attention. This changed in 2013, when a local groundwater-based waterworks (Brantafors in Kallinge, Ronneby Municipality) had to close due to PFAS concentrations of up to 10 000 ng/L in outgoing drinking water (Jakobsson et al. 2014). There were earlier incidents in Sweden: the waterworks in Tullinge (Botkyrka municipality) was closed in 2011 due to high PFOS concentrations (Swedish Chemicals Agency 2013), and in Uppsala PFAAs—amongst others PFOS and PFOA—were detected in 2012 in drinking water (Gyllenhammar et al. 2015). In order to minimize exposure of the population to PFASs via drinking water, the waterworks in Uppsala were equipped with carbon filters. However, it was only after the shutdown of the waterworks in Brantafors that significant public concern arose, and media attention developed. PFASs are now seen as “one of the most serious chemical disasters in Sweden for a very long time” (Bergman et al. 2014).

As a result, nationwide screening for PFASs at waterworks was initiated and is ongoing until the end of 2016 (Livsmedelsverket 2014c, d). The Swedish Water and Wastewater Association (Svenskt Vatten) conducted a survey on PFAAs in raw and drinking water among its members in 2014 (Holmström et al. 2014), see “Occurrence” section. There are also currently investigations of the situation in groundwater and surface water in connection to some of the point contaminated areas, usually conducted by the problem owners (The Swedish Armed Forces, commercial airport companies, and related organizations), see also “Occurrence” section. Another investigation assigned by the Swedish Environmental Protection Agency on PFASs in surface and groundwater was realized in 2015 (Ahrens et al. 2016; Naturvårdsverket 2016), see “Occurrence” section.

Groundwater contamination of PFASs has not been investigated in the same systematic way as surface water contamination. Groundwater samples are usually only representative for the immediate vicinity of the well where the sample was taken. A representative spatial screening would therefore require a very large number of samples to be taken. As this is not feasible, groundwater sampling has mainly focused on known or suspected PFAS hotspots at airfields (Filipovic et al. 2015b) and landfill sites (Ahrens et al. 2016).

### Occurrence

In the survey by the Swedish Water and Wastewater association (Holmström et al. 2014, see also “Monitoring history” section), water samples were analyzed for the 7 PFAAs that were then mandated for monitoring by the National Food Agency (*note: as of today this number has risen to* 11 *PFASs, see* “Legal situation” *section on the legal situation*). The collected dataset represents the water production for 4.3 million end consumers in Sweden. In total, 22% of all samples (52 out of 236) contained PFASs in detectable amounts. The detection frequency in samples from surface water supplies was much higher than in those from groundwater (Table 1), and PFOS and PFOA were the compounds most frequently detected. Distinct values for PFAAs concentrations in drinking water for some Swedish municipalities are also presented in several risk assessments of the National Food Agency (Livsmedelsverket 2013, 2014b). Reported values for raw drinking water in these studies reach up to 4000 ng/L for PFOS, and up to 130 ng/L for PFOA.

**Table 1:** PFAAs detection frequency in raw and drinking water samples from different water supplies in Sweden

Type of water supply	Analyzed water supplies (*n*)	Detection frequency PFAAs (%)
Surface water	27	37
Artificial recharge	12	50
Groundwater	193	19

Holmström et al. (2014), detection limit for individual species: 1 or 2.5 ng/L

In the screening of groundwater, surface water sewage treatment plant (STP) effluents, and landfill leachates achieved by Ahrens et al. (2016)—see also “Monitoring history” section on monitoring history—502 water samples were analyzed for 26 PFASs. Samples originating from drinking water source areas had an average sum of all 26 PFASs of 8.4 ng/L. Of these, 2% were above the threshold value for the (then) 7 PFAAs as recommended by the National Food Agency of 90 ng/L. Average concentrations for the sum of the 26 analyzed PFASs were 487 ng/L (landfill leachates), 112 ng/L (surface water), 49 ng/L (groundwater), 35 ng/L (STP effluents), and 3.4 ng/L (background screening lakes). The high PFAS concentrations in landfill leachates are of concern, as landfill sites usually do not focus on the removal of PFASs, which makes them act as potential hotspots of PFASs to surface and groundwater.

Groundwater samples taken directly at firefighting training areas at commercial airports show PFOS concentrations between 2700 and 2 910 000 ng/L (Wennberg and Fridlund 2015), while measurements in groundwater at Tullinge yielded PFOS concentrations of up to 42 200 ng/L, and PFOA concentrations up to 4470 ng/L (Filipovic et al. 2015b). The project RE-PATH addressed the PFAS problem at airports in Sweden between 2009 and 2014 (see final report, Norström et al. 2015). The project concluded that drinking water around the airports Arlanda (Stockholm) and Landvetter (Göteborg) is not threatened by contaminated groundwater as no large public waterworks are close by. However, they go on to state that assurances are needed to safeguard that the identified contaminated areas will not pose a risk for drinking water in the future. Although drinking water may not be at risk, EU law effectively prohibits input into groundwater of any organohalogens, which include PFASs (EU directives 2000/60/EC and 2006/118/EC).

While the study by Holmström et al. (2014) suggests that groundwater is less contaminated than surface water or areas of artificial recharge, groundwater is still significantly affected by PFASs. In fact, the—by far—highest PFAS concentrations are found in groundwater, especially in close proximity to point sources such as firefighting training areas at airports (Wennberg and Fridlund 2015). This is of concern as 50% of Sweden’s drinking water comes from groundwater (Svenskt Vatten 2000), and many aquifers that provide it are located in areas of sand and gravel deposits. The corresponding high hydraulic conductivity of these areas allows the extraction of large volumes of water, but also for the rapid spreading of dissolved contaminants, such as PFASs, over large areas, complicating cleanup efforts. However, even in lower permeability geological deposits, PFAS contamination is a problem as the combination of long groundwater residence times and persistence of most PFASs results in a long-time presence of these compounds in our water resources, see also (Cousins et al. 2016).

### Legal situation

Over a decade ago, the Swedish Chemicals Agency conducted a risk assessment for PFOS (Swedish Chemicals Agency 2004a, b) and raised concerns about its presence in Sweden. It even recommended the ban of PFOS in the country. Despite this, firefighting foams—including PFOS—were finally phased out between 2003 and 2011 (Berglind et al. 2013) and it has only been within the last 2 years that Swedish authorities have established threshold values for different types of waters.

Today, there exists an action limit for the sum of 11 PFAS compounds in drinking water of 90 ng/L in Sweden, provided by the National Food Agency (Livsmedelsverket 2016), including: perfluorobutane sulfonate (PFBS), perfluorohexane sulfonate (PFHxS), PFOS, 6:2 fluorotelomer sulfonic acid (6:2 FTSA), perfluorobutanoic acid (PFBA), perfluoro-n-pentanoic acid (PFPeA), perfluorohexanoic acid (PFHxA), perfluoroheptanoic acid (PFHpA), PFOA, perfluorononanoic acid (PFNA), and perfluorodecanoic acid (PFDA). This action limit is based on a potential risk for human health coming from PFASs in drinking water, for details see Livsmedelsverket (2014a). If concentrations of these 11 compounds are higher than the action limit, measures need to be taken in order to reduce them. Until March 2016, this list contained only 7 PFAS compounds (PFBS, PFHxS, PFOS, PFPeA, PFHxA, PFHpA, PFOA), but was extended.

Moreover, the Swedish Geotechnical Institute (SGI) was appointed by the government to suggest a preliminary PFOS threshold value for soil and groundwater, and the values landed on 45 ng/L for groundwater and on 0.003 mg/kg for sensitive land use, in order to protect Swedish natural resources (Pettersson et al. 2015). Furthermore, PFASs have an impact on at least three of Sweden’s environmental objectives, namely, *a Non*-*Toxic Environment*, *Flourishing Lakes and Streams,* and *Good*-*Quality Groundwater* (Naturvårdsverket 2015b). In the recent report of the Swedish Environmental Protection Agency on these goals, the goals of *a Non*-*Toxic Environment and Good*-*Quality Groundwater* are named as not achievable until 2020, and PFASs are explicitly named as one problematic issue (Naturvårdsverket 2015a).

The Swedish Environmental Code (Miljöbalken) espouses the polluter pays principle. The legal situation can therefore have significant financial implications for stakeholders. In particular, if a groundwater body that possesses a legal ID from one of the five water authorities (Vattenmyndighet) in Sweden becomes contaminated, European Union (EU) law takes effect according to Groundwater Directive 2006/118/EC. Article 4 of this directive requires that member states shall “take such measures as may be necessary to protect aquatic ecosystems, terrestrial ecosystems and human uses of groundwater dependent on the part of the body of groundwater represented by the monitoring point or points at which the value for a groundwater quality standard or the threshold value has been exceeded.”

The presence of PFASs is a clear case of an anthropogenic impact that invokes the necessity to protect the affected groundwater resource. PFAS contamination of several groundwater bodies used for drinking water extraction has led to recognition by the water authorities leading to increased legal protection.

While the water authorities have the mandate to identify legally protected groundwater bodies, it is the regulatory authorities (tillsynsmyndighet) that have oversight over individual cases of PFAS contamination and mandate if any remedial action is required. In most cases, the regulatory authority is the county (Länsstyrelsen) or municipality (kommun). Water authorities do not have a direct regulatory function; they are primarily responsible for coordinating water-related activities at the catchment scale, which may extend over political boundaries. The regulatory authority—and not the water authority—is the only body that can make legally binding decisions that consider site-specific issues. These decisions can include legally binding obligations to remediate, and they may be appealed and overturned by the Land and environmental courts (Mark- och miljödomstolen).

To date, there is very little case law dealing with PFAS contamination in particular and contaminated groundwater in general. The regulatory authorities therefore have little to base their decisions on beyond the Environmental Code and the EU directive—both of which can be deliberately vague. The costs associated with remedial action for PFASs are high and are likely to be contested. This means that further direction may soon be available in the form of legal precedents.

### Remediation actions taken

Most of the sites in Sweden affected by point sources have not been investigated and even less, remediated. Notwithstanding, the Swedish Civil Contingencies Agency’s firefighting training area at Rosersberg has been remediated with a focus on excavating soil masses affected by oil spill, but not with respect to PFASs. Currently, investigations are proceeding on the PFAS contaminant situation, and discussions on replacing the heavily contaminated drainage pipes are ongoing (Karlsson, SGU 9 Feb 2016, pers. comm.).

At Malmö Sturup, Göteborg Landvetter, and Stockholm Arlanda airports, as well as the former military airfield F18 Tullinge, Stockholm, the remediation actions taken for the PFOS-contaminated firefighting training areas consist of collecting storm-, surface- and groundwater, cleaning via activated carbon filters (Norström et al. 2013; Woldegiorgis 2015). Different reports give various indications on the results of this filtering action, where the cleaning degree given is large (>99%), but with such a small rate of remediation at 0.2 m^3^/h (Woldegiorgis 2015), whereby remediation times become unreasonable. Natural transport out of the affected areas is much larger than the volumes cleaned on site. The current remediation actions have primarily focused on contaminated groundwater using ‘pump and treat’; there is a clear need of investigations of how to remediate the hotspot areas with focus on the unsaturated zone.

## Current developments and future challenges

### Current analytical technology development

Historically, the major challenges with the analysis of PFASs in environmental samples have been primarily technical, as there was a lack of isotopically labeled standards and LC-MS/MS instruments were not very sensitive. The first studies in the early 2000s were reporting mainly two PFASs: PFOA and PFOS (Giesy and Kannan 2001). However, over the last 15 years huge advances in detection technology have been achieved. Today, there are numerous isotopically labeled standards commercially available, and advances in mass spectrometry have lowered the instrumental detection limits from µg/L to ng/L. New instruments can reach down to pg/L which is more than three orders of magnitude decrease in response. This allows investigators to report PFAS concentrations below previous detection limits (Vestergren et al. 2012; Filipovic et al. 2015a). Today, a suite of different PFAS subgroups (including 10–20+ individual compounds) is often reported in the scientific literature (Ahrens and Bundschuh 2014). As the number of analyzed PFASs increases beyond only PFOA and PFOS in recent scientific literature, this allows a broad range of PFASs to be compared between different studies, giving a better understanding of current production, use, and spreading of PFASs around the world.

PFAS precursors contain moieties, which can be transformed in the nature and form persistent PFASs such as PFOA and PFOS. PFAS precursors based on shorter chain chemistry (C4–C6) are currently replacing longer chain PFASs, such as PFOA and PFOS, in consumer products. A technical challenge is how to identify and analyze PFAS precursor compounds. Historically, longer chain PFASs and PFAS precursors were used as the main components in AFFF; today, longer chain PFAS have been replaced by 6:2 fluorotelomers which can—when released into the environment—transform into shorter chain PFAAs (Kim et al. 2014). Most of the PFASs being reported in groundwater from Sweden have primarily been PFCAs and PFSAs with a limited number of PFAS precursors following the regulation of the Swedish authorities (Ahrens et al. 2015; Filipovic et al. 2015a). As a result of the increasing awareness regarding the problem with PFASs in Swedish waters, methods covering a broader spectrum of PFASs have newly been developed. In a recent study by Ahrens et al. (2016), 26 PFASs were monitored, including 9 PFAS precursor compounds. Some precursor compounds were also found in groundwater (perfluorooctane sulfonamide, FOSA, and 6:2 FTSA) and are therefore suggested to be included within the drinking water guidelines (Ahrens et al. 2016).

Among the PFAS precursor compounds analyzed in AFFF used in Sweden, 6:2 FTSA is frequently detected in high concentrations (Swedish Chemicals Agency 2015a) and AFFF contaminated areas (Regeringskansliet 2016). Although studies suggest that another precursor—6:2 fluorotelomer sulfonamide betaine (6:2 FTAB)—exists, there is only a limited number of studies where 6:2 FTAB has been analyzed and quantified in environmental samples (Moe et al. 2012; Boiteux et al. 2016; Munoz et al. 2016). Furthermore, over a dozen not frequently analyzed PFASs and PFAS precursor compounds have been identified in AFFF used in the USA, among them 8:2 FTSA (fluorotelomer sulfonic acid), zwitterionic PFASs, and shorter chain PFASs (C2–C3) (Backe et al. 2013; Barzen-Hanson and Field 2015). The Swedish Geotechnical Institute (SGI) has recently suggested a ban of PFAS-based AFFF except in situations where PFAS-based AFFF are required (SGI 2016).

The Swedish guideline values for PFASs in drinking water do not include the possible presence of precursor compounds (Livsmedelsverket 2016). As there is still the technical issue of identifying and quantifying the numerous known and unknown PFASs in the environment, novel approaches to address this issue have been developed. The first approach is the so-called total precursor assay (TOP-assay), where oxidizing agents are used to degrade all potential PFAS precursors to form perfluoroalkyl acids as end products (Houtz et al. 2013). Using the TOP-assay on water samples has shown that there might be an increase of the measured perfluoroalkyl acids of up to 100%. The amount of perfluoroalkyl acids produced is hypothetically equivalent to the total concentration of PFAS precursors in the samples. A second approach is to measure the total organic fluorine in environmental samples. This method can be done with combustion ion chromatography (CIC) (Weiner et al. 2013), or particle-induced gamma emission spectroscopy (PIGE), allowing for the measurement of total organic fluorine atoms in environmental samples (Hashiguchi et al. 2013). Using a combustion-IC, all substances containing fluorine (i.e., both organic and inorganic) are converted to hydrogen fluoride (HF), which is subsequently trapped in a water-filled absorption unit, wherein HF dissociates to H^+^ and F^−^ ions. This solution is then injected onto the ion chromatograph (IC). Importantly, if the fluorine signal is suspected to be coming from inorganic fluorine in the sample, this can be tested either directly using the IC (i.e., without combustion) or by solid-phase extraction followed by CIC (Miyake et al. 2007a, b). In general, TOF methods have generally lower sensitivity, leading to higher limit of quantifications (LOQ) compared to conventional LC-MS/MS methods. The limitation of TOF-analysis is that it only provides a measurement of total fluorine; nevertheless, the simplicity and no sample preparation makes TOF a rapid screening technique, which captures all PFASs. However, CIC analysis requires proper sample preparation where Teflon (PTFE) parts should be avoided. Otherwise, impurities of PTFE (which has high fluorine density) might bias the fluorine signal. The results from TOF measurements are further used to calculate hypothetical PFOS/PFOA equivalents in the sample. Measuring total organic fluorine is often conducted simultaneously to conventional PFAS analysis. A combination of these two methods makes it easier to evaluate the amount of “unknown PFASs” in the sample (Hashiguchi et al. 2013; Weiner et al. 2013). To identify PFASs, which have previously not been ‘identified,’ the development of characterization techniques using high-resolution LC-MS/MS has been assessed, finding PFASs previously not reported (Munoz et al. 2016). To date, there are no commercial or university laboratories in Sweden offering TOP-assay, which is problematic as human beings might be exposed to far higher amounts of PFASs than current analytical techniques are able to report. The novel analytical methods, such as the CIC and TOP-assay, are eventually a necessity in order to analyze and quantify total PFAS amounts in drinking water samples. Without the TOP-assay or CIC, the guidelines of PFASs in drinking water are lacking insight into numerous non-frequently analyzed PFAS precursors, which can transform to ultimate persistent PFASs and their abundance in the aquatic environment. Using the TOP-assay on water samples has shown that there might be an increase of the measured perfluoroalkyl acids of up to 100%. While showing great promise across a wide range of matrices, the TOP-assay in its current state has several limitations. First, the analytical method has not been evaluated for a wide range of PFAS precursor compounds. Second, there is a lack of information regarding the chemical oxidation efficiency with presence of co-contaminants and organic carbon in the matrix. Third, the oxidation process is hard to control leading to mixed results where some PFASs (i) do not fully oxidize during the TOP-assay and (ii) some PFASs mineralize. This makes the TOP-assay a qualitative method for determination of PFAS precursors; however, the quantification of the results remains still problematic. Fourth, the analytical method described by Houtz and Sedlak (2012) can only measure from perfluorobutanoic acid (C4) through to perfluorotetradecanoic acid (C14). However, ultra-short chain (i.e., <C4) or very long-chain (i.e., >C14) PFAAs may also form following oxidation of some PFAS precursors which are not measured with current analytical methods. In order to maximize coverage of PFAA chain lengths, development of chromatography methods covering a broader range of PFAS C2–C18 PFCAs should be developed and applied analogous with the TOP-assay. The limitations listed above show that the TOP-assay is currently not easy to standardize.

### Future challenges

There are several major challenges that will have to be dealt with when it comes to the environmental damage being caused by PFASs:

1. As a result of the low level of communication between Swedish authorities (Regeringskansliet 2016), and the lack of hard threshold values for PFASs (which is partly being addressed now, see “Legal situation” section), municipalities do not know how to react and which concrete measures should be taken if groundwater, or even drinking water, is found to be contaminated with PFASs. Clear guidelines and procedures have to be developed and established countrywide to solve this unclear situation.
2. There are too many authorities in Sweden with part-responsibilities for Sweden’s water resources (Lewis et al. 2013), resulting in important issues being overlooked and neglected (Regeringskansliet 2016).
3. Presently, the Swedish monitoring/screening strategy for PFASs still focuses on contamination hotspots, which is a general preference in Sweden and neglects diffuse sources from previous and ongoing pollution inputs (Baresel et al. 2006; Destouni et al. 2010). However, contamination has been ongoing for decades, and that means contaminants have had time to move long distances. This needs to be taken account of.
4. There are large groundwater monitoring gaps in Sweden like neglected pollution contributions that have to be taken into account (Destouni et al. 2008; Baresel and Destouni 2009).
5. The infiltration into groundwater from contaminated sites has to be reduced to avoid further contamination of groundwater with PFASs. This is needed as PFASs that are pooled in the unsaturated zone will continue to infiltrate and spread from contaminated areas as long as the source is not removed, or infiltration of precipitation is inhibited.
6. As of today, 25% of all drinking water extraction sites in Sweden lack a protection area, and 60% of the existing protection areas are old and poorly constrained, therefore needing revision. This situation makes it difficult to adequately protect drinking water sources from PFAS contamination.
7. The ~800 000 private drinking water wells in Sweden, which provide for 1.2 million permanent residents, and about the same number of temporary ones (Socialstyrelsen 2008) have to be included in the monitoring as they lack any protection and control.
8. Strategies for how to deal and clean up PFAS leakage from landfills need to be dealt with, as this type of source contributes to high levels of PFASs according to the investigation performed by Ahrens et al. (2016). More sampling and monitoring is needed to get an overall picture of the situation. WWTPs need better treatment of PFASs and to initiate the development thereof, legal initiatives with guideline values for accepted levels of emissions are needed.
9. There are currently no reasonable in situ or ex situ remediation techniques available that can deal with PFAS cleanup on the scale required for aquifers or watersheds. This is an area that requires a great deal of fundamental research and no easy solutions on the question ‘how to clean up?’ are on the horizon.
10. The even greater challenge is to ban production and use of the harmful PFASs, and to find sustainable and non-harmful replacements. The PFAS group is enormous (more than 3000 different substances may have been in use, Naturvårdsverket (2016)) and when authorities ban one, producers change to another closely related substance with properties like the first one (Gomis et al. 2015). This behavior needs to be stopped and a better communication between researchers, authorities, and producers is of uttermost importance.
11. Last but not least, funding for all the above points is needed and this can be achieved in different ways. A water tax could be enforced in Sweden as we have a very cheap tap water financing the work concerning water protection and monitoring, e.g., as done in Denmark. Polluters pay principle should be enforced for the cleanup of contaminated sites. Higher costs for the registration of chemicals and higher demands on pre-investigations of the impacts of a chemical on human health and the environment, this to make sure only safe and highly needed chemicals enters the market. Demands on the producers to make analytical techniques publically available to lower costs for monitoring.

Many of the challenges listed here can be extended to other problems regarding (ground) water contamination, which have received little attention so far, such as pesticides, chlorinated solvents, corrosion inhibitors, and pharmaceuticals. The PFAS problem and the shortcomings when it comes to groundwater quality monitoring are exemplary for the general monitoring situation in Sweden, which was criticized by the EU commission (European Commission 2012) and that urgently should be improved. As stated above, there are too many authorities partly responsible for monitoring today and coordination is highly insufficient (Åkesson et al. 2015; Augustsson et al. 2016). One single authority should be designated to have the overall responsibility for water monitoring, which was also suggested by a recent governmental report (Regeringskansliet 2016). This means that the issue needs to be lifted high on the political agenda. Possible financial instruments could be a better implementation of the rarely applied *Polluter Pays Principle,* or the introduction of fees or taxes for the release of emissions and water takeout, e.g., as applied in Sweden’s neighboring country Denmark (20–40 cents/m^3^). In many other countries, fees and/or taxes pay for mapping, monitoring, and the necessary measures to be taken in order to ensure good-quality water resources.

## Conclusions

PFASs have been detected in both raw and drinking water in Sweden, thus potentially affecting the drinking water for more than 3.6 million inhabitants. Some sources, i.e., firefighting training areas, have been identified and the monitoring of groundwater, surface water, and drinking water is ongoing. However, much is left to do in order to obtain a sound understanding of the current situation in Sweden. The situation is even more unclear when it comes to potential measures that should be realized in the future in order to handle PFAS contamination of raw and drinking water in Sweden in a meaningful way. A prerequisite for this is the development of a sound strategy on how to identify, investigate, and monitor PFAS-contaminated sites on a national scale.

Finally, the authors would like to express their concerns that the protection of groundwater, the world’s and even Sweden’s largest and most reliable source of freshwater, is not receiving adequate attention today. Society should be aware that the groundwater formed today will be consumed by our children, grandchildren, and future generations. Thus, it is urgent to address the issue of good-quality groundwater and safe future drinking water.
